# Assessing Oncologists’ Attitudes Concerning Comprehensive Genomic Profiling in Stage IV Lung Adenocarcinoma in Brazil

**DOI:** 10.1016/j.jtocrr.2022.100402

**Published:** 2022-08-30

**Authors:** Aline F. Fares, Pedro H. Martinez, Pedro H. Farina, Isaac Bicalho de Souza, Daniel V. Araújo, Narayana S. Paiva, Ligia F. Orlando, Tatiana Elias Colombo, Eldsamira Mascarenhas, Ana Caroline Z. Gelatti, Clarissa Baldotto, Mauro Zukin, Luiz Henrique Araujo, Clarissa Mathias, Gustavo Werutsky, Gilberto de Castro, Vladmir C. Cordeiro de Lima

**Affiliations:** a Department of Medical Oncology, Hospital de Base de São José do Rio Preto and Faculdade de Medicina de São José do Rio Preto, São José do Rio Preto, SP, Brazil; b Brazilian Group of Thoracic Oncology - GBOT, Salvador, Brazil; c Rede D’Or Oncologia Bahia, Salvador, Brazil; d Oncoclínicas Rio Grande do Sul, Porto Alegre, Brazil; e Instituto D'Or de Pesquisa e Ensino, Rio de Janeiro, Brazil; f DASA Oncologia e Genômica, Rio de janeiro, Brazil; g Cancer National Institute (INCA), Rio de Janeiro, Brazil; h Oncoclínicas Bahia, Salvador, Brazil; i Latin American Clinical Oncology Group, Porto Alegre, Brazil; j Department of Medical Oncology, Institute of Cancer of São Paulo– ICESP, Faculty of Medicine of the Universidade de São Paulo, SP, Brazil; k AC Camargo Cancer Center, São Paulo, Brazil

**Keywords:** Oncogenic-driven, Lung, Adenocarcinoma, Comprehensive genomic testing

## Abstract

**Introduction:**

Advances in comprehensive genomic profiling (CGP) of lung adenocarcinomas (LUADs) led to personalized treatment for patients. This study evaluated medical oncologists’ attitudes toward CGP in a scenario where sponsored funding for CGP was available.

**Methods:**

We designed an online survey assessing CGP use and treating physicians’ confidence, composed of three self-confidence domains, which are as follows: confidence in interpreting CGP results, confidence in treating oncogenic-driven LUAD, and confidence in managing tyrosine kinase inhibitor adverse events. The survey was distributed to medical oncologists who treat lung cancer in Brazil. Comparisons between groups were performed using the chi-square or Fisher’s exact test. Univariable and multivariable (adjusted OR) analyses were performed.

**Results:**

Among 104 respondents who treat patients with lung cancer, 55% were from the Southeast region, 28% had high lung cancer clinical load, and 33% had in-house molecular testing. More than half (51%) of the participants request CGP systematically to stage IV LUAD. As for provider confidence, 67% stated being confident in all three domains: 76% confident in interpreting CGP, 84% confident in treating oncogenic-driven LUAD, and 81% in managing tyrosine kinase inhibitor adverse events. Providers’ confidence was associated with systematically requesting CGP to stage IV LUAD (*p* = 0.013). After controlling for the variables of interest, systematic requesting CGP for stage IV LUAD revealed a significant association with the provider’s confidence (adjusted OR = 0.35, *p* = 0.028, 95% CI: 0.14–0.84). The major challenge for properly requesting CGP was the long turnaround time and the fear of treatment delays.

**Conclusions:**

Even though CGP for stage IV LUAD in Brazil is fully sponsored, only half of the oncologists in our survey systematically request it.. Requesting CGP was associated with providers’ confidence. Improving access and promoting providers’ awareness of CGP utility is necessary to increase CGP use and better inform treatment decisions.

## Introduction

The landscape of lung cancer has changed dramatically in the past decade. The availability of molecular profiling yielded valuable information for treatment and prognosis.^1^ If properly sequenced, approximately 60% of lung cancers will contain a genomic driver. Targeted therapies guided by genomic drivers improve survival and are associated with lower toxicity, ultimately improving patients’ quality of life.^1^^,^^2^

Recent guidelines recommend comprehensive genomic profiling (CGP) of all patients with stage IV nonsquamous, nonsmall cell carcinomas of the lung, represented mainly by lung adenocarcinomas (LUADs). They also state that CGP should be considered for squamous cell histology, especially for never smokers.^3^, ^4^, ^5^, ^6^ Testing results should be available before initiating systemic therapy to guide first-line treatment properly. As of this writing, the Food and Drug Administration has approved targeted therapies for *EGFR*, *ALK*, *ROS1*, *KRAS G12C*, *BRAF V600E*, *MET*, *RET*, and *NTRK* alterations; thus, LUAD samples should ideally have this minimum of eight genes appropriately sequenced.^1^ Nevertheless, fewer than half of patients have their tumors tested for the four most often altered genes (*EGFR*, *ALK*, *ROS1*, and *BRAF*) in LUAD.^7^ Numbers are even smaller when analyzing tumors sequenced before any systemic therapy.^7^

The barriers to proper sequencing have been a matter of extensive discussion.^8^^,^^9^ They include sequencing costs, tissue quality and availability, long turnaround time, lack of access to treatments and clinical trials, and provider lack of awareness. In Brazil, a consortium of biopharmaceutical companies named LungMapping funds FoundationOne (Foundation Medicine, Inc; Foundation Medicine) testing for patients with stage IV LUAD, improving access to CGP in these cases.^10^ Blood-based sequencing (through sequencing of circulating tumor DNA) is also helping to overcome some of these challenges, particularly the lack of available tumor specimens.^8^ With the increasing number of patients with lung cancer submitted to blood and tissue sequencing, providers should have a basic understanding of genomics.^8^ Few studies have evaluated providers’ attitudes toward genomic findings. Nevertheless, recent evidence has revealed a lack of confidence among providers in using genomic sequencing for malignancies, possibly limiting its benefits in clinical practice. Given the increased complexity of LUAD treatment after the advent of new sequencing technologies, complex biomarkers, and new study designs, providers must understand how to interpret and incorporate genomic findings to help the individual workup and, ultimately, the decision-making process.

Here, we aim to evaluate how frequently medical oncologists request CGP for stage IV LUAD and how this correlates with their confidence in managing oncogenic-driven LUAD. We hypothesize that confident providers managing oncogenic-driven LUAD have higher chances of systematically requesting CGP. Moreover, we investigate individual- and practice-level characteristic details of providers’ challenges in requesting CGP and explore the features of those hesitating to request it.

## Materials and Methods

### Study Design

This study used a web-based survey to analyze broad genomic sequencing for LUAD in Brazil. It was supported by the Brazilian Group of Thoracic Oncology (Grupo Brasileiro de Oncologia Torácica [GBOT]) and the Latin American Cooperative Oncology Group. This cross-sectional study was approved by the Faculty of Medicine of São José do Rio Preto Research Ethics Board.

### Survey Design

Two thoracic medical oncologists designed the survey (AFF and VCCL), and, sequentially, it was approved by GBOT and the Latin American Cooperative Oncology Group. This is an anonymous questionnaire consisting of 13 questions. The estimated time to completion of the survey was 10 minutes. We used Google Forms software to create the survey.

The primary objective was to describe the relationship between providers’ confidence and CGP requests. Secondary objectives included (1) to profile medical oncologists treating lung cancer in Brazil, including the region of the country where they practice, the number of patients with lung cancer they see monthly, the number of patients with lung cancer with *EGFR/ALK* alterations under their care, and in-house genomic testing availability; (2) to evaluate medical oncologists’ confidence to interpret CGP; and (3) to evaluate medical oncologists’ confidence to treat driver-positive LUAD and to manage tyrosine kinase inhibitor (TKI) adverse events.

We queried participants about their intentions to request CGP for patients with stage IV LUAD in a percentage manner. We created a providers’ confidence variable by combining the following three domains: (1) providers’ confidence in interpreting CGP; (2) providers’ confidence in treating oncogenic-driven LUAD; and (3) providers’ confidence in managing TKIs’ adverse events. The confidence level was scored 0 to 10 regarding treatment and managing side effects. Confidence in interpreting genomic tests was considered as confident or not confident. The variable providers’ confidence was categorized as “not confident”—if any of the three domains were reported as not completely confident—or “confident” if all three domains were registered as entirely confident.

We considered the variable “number of lung cancer patients seen weekly” as a surrogate to define oncologists practicing with a focus on lung cancer (versus general oncologists) if they see more than 10 patients with lung cancer weekly. We used a surrogate to mitigate the risk of tracking back the provider on the basis of the reported characteristics, increasing the accuracy of the reported information. The survey instrument is included in the Supplementary Data.

### Participants’ Selection and CGP Testing Availability in Brazil

There are currently 350 physicians registered in GBOT. This total includes medical oncologists and physicians in other specialties practicing lung cancer. Therefore, to e-mail the survey, we excluded all non-oncologists and oncologists practicing abroad. Finally, the survey was e-mailed to 287 oncologists practicing in Brazil and registered in GBOT. To maintain the anonymity of the participants, we have not provided physicians with financial incentive nor verified if the survey had been received. We were concerned that breaking anonymity would discourage participants from recording their confidence in managing lung cancer. After we received the responses, we excluded providers reporting not treating patients with lung cancer from the final analysis.

Regarding CGP testing in Brazil, various platforms are available, and pharmaceutical companies supply most tests. Since 2019, the industry-sponsored program, LungMapping, provides access to FoundationOne testing of confirmed LUAD in the public and private health care settings throughout the country. Turnaround time after shipping is 3 weeks, and the test is performed in the United States.

### Data and Statistical Analysis

Descriptive statistics were generated, including median and means for continuous variables and proportions for categorical variables. We used the chi-square or Fisher’s exact tests to investigate associations between qualitative variables, using a statistical significance α level of 0.05. Owing to the low number of participants in some Brazilian regions (particularly the North region), we merged the South and Southeast regions and North, Northeast, and Mid-West regions to perform comparisons.

Providers were asked about confidence in the following three domains: interpreting genomic test results, treating driver-positive LUAD, and managing TKIs’ adverse events. The first domain, referring to the interpretation of genomic test results, was evaluated as confident versus not confident. The second and third domains were each scored 0 to 10 by the participants. We considered a cutoff greater than or equal to 8 to be reasonable good self-confidence scoring and therefore defined it as confident in treating and managing driver-positive LUAD adverse events.

We performed univariable and multivariable analyses to understand how medical oncologists’ confidence performs after adjusting for variables of interest in clinical practice. The following variables were selected for multivariable regression: Brazilian state of practice (São Paulo versus others), number of patients with lung cancer seen weekly (≥10 patients versus <10 patients), time practicing oncology (≥10 y versus <10 y), to order CGP to all stage IV LUAD (yes versus no), in-house genomic testing available (yes versus no), level of confidence treating driver-positive lung cancer (≥8 versus <8), and level of confidence managing TKIs’ adverse events (≥8 versus <8). We used SPSS version 27^11^ to perform statistics tests.

## Results

### Participant’s Characteristics

A total of 106 medical oncologists responded to the online survey. Although we specified that the study was dedicated to medical oncologists treating lung cancer, two participants responded as “not treating lung cancer” and were therefore excluded from the analysis, with a final number of 104 participants.

**Figure 1:**
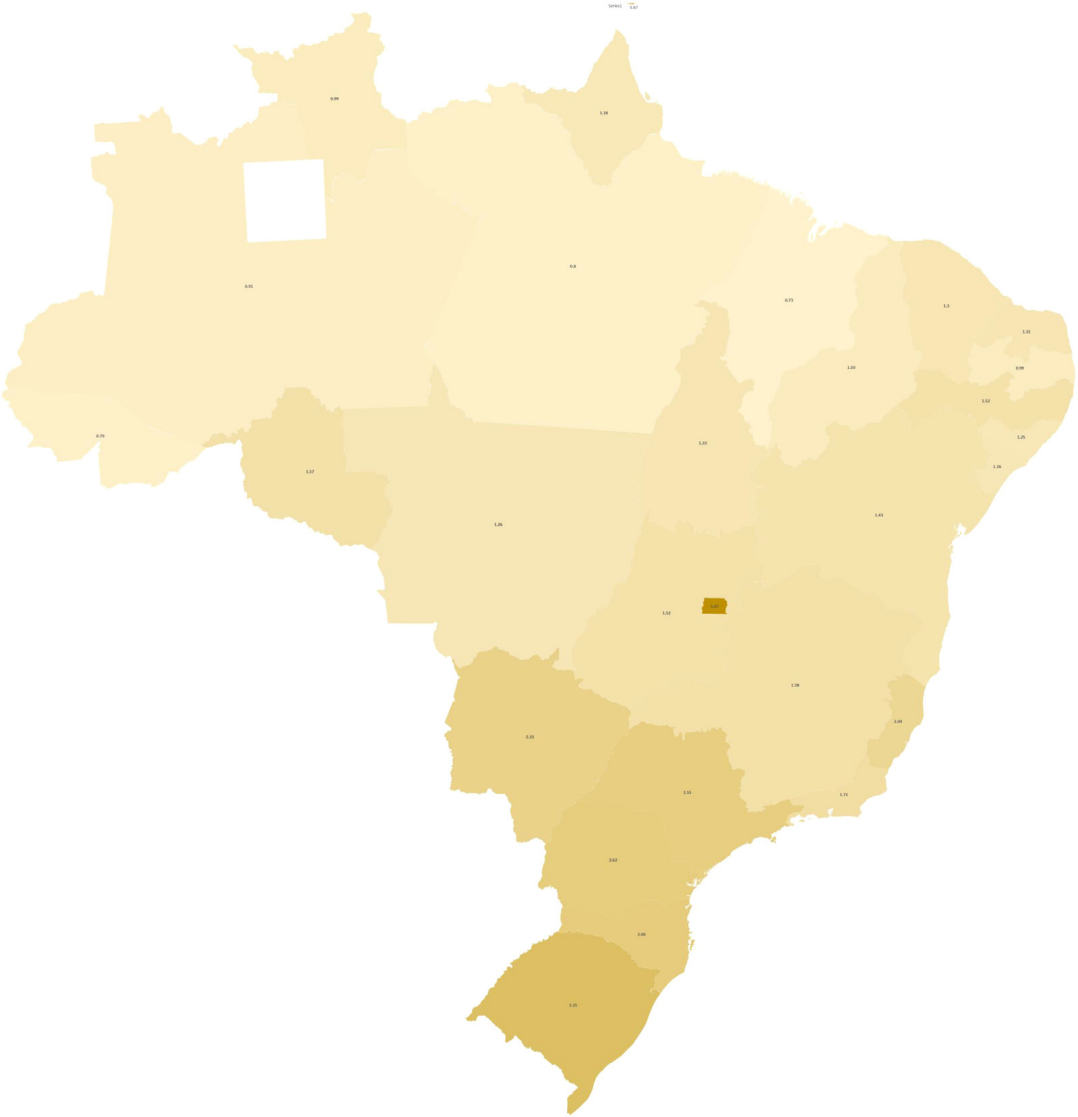
Brazil map illustrating the number of medical oncologists per 100,000 Brazilians. The dashed line reveals the limits separating Southeast and South Brazil from other regions. Adapted from Scheffer, M. et al., Demografia Médica no Brasil 2020. São Paulo, SP: FMUSP, CFM, 2020. 312 p. ISBN: 978-65-00-12370-8.

Participants were based in all regions of Brazil, including North, Northeast, Mid-West, Southeast, and South. Most participants were established in the Southeast region (57 medical oncologists, 54.8%), and the least represented was the North region (two medical oncologists, 1.9%). These data are consistent with Brazil’s medical oncologist density (Fig. 1). Of the participants, 72% practice as general oncologists, seeing less than 10 patients with lung cancer weekly. There was no association between the region of Brazil and the number of patients with lung cancer seen weekly (*p* = 0.42), revealing throughout the country, most oncologists practice as general medical oncologists, regardless of their state or region. Similarly, there was no association between the number of patients with *EGFR* or *ALK* alterations seen and the country’s region (*p* = 0.27 and *p* = 0.37, respectively). There were 59 participants (56.7%) who reported less than 10 years of experience in oncology.

### Provider’s Level of Confidence

Providers were asked about confidence in the following three domains: interpreting genomic test results, treating oncogenic-driven LUAD, and managing TKIs’ adverse events. Approximately 67% of the respondents stated confidence in all three domains. Of the remainder of the respondents, 6% lacked confidence across all domains, 24% lacked confidence in interpreting genomic test results, 15% in treating oncogenic-driven LUAD, and 18% in managing TKIs’ adverse events. Details are found in Table 1.

**Table 1:** Baseline Characteristics of Respondents

Variable of Interest	Categories	Total, N (%) 104 (100)	How Frequently Do You Order CGP to Stage IV LUAD?		*p* Value
			Always n = 53 (51%)	Not Always n = 51 (49%)	
Region practicing oncology					
	Mid-west	6 (5.8)	2 (3.8)	4 (7.8)	0.28
	Northeast	23 (22.1)	11 (20.8)	12 (23.5)	
	North	2 (1.9)	1 (1.9)	1 (1)	
	South	16 (15.4)	5 (9.4)	11 (21.6)	
	Southeast	57 (54.8)	34 (64.2)	23 (45.1)	
Years practicing oncology					
	<10 y	59 (56.7)	29 (54.7)	30 (58.8)	0.41
	≥10 y	45 (43.3)	24 (45.3)	21 (41.2)	
Number of patients with lung cancer seen weekly					
	<10	75 (72.1)	38 (71.7)	37 (72.5)	0.54
	≥10	29 (27.9)	15 (28.3)	14 (27.5)	
Current *EGFR*-mutant clinical load					
	<10	84 (80.8)	16 (30.2)	4 (7.8)	0.001
	≥10	20 (19.8)	37(60.8)	47 (92.2)	
Current *ALK*-positive clinical load					
	<3	80 (76.9)	36 (67.9)	44 (86.3)	0.023
	≥3	24 (23.1)	17 (32.1)	7 (13.7)	
In-house testing available					
	Yes	34 (32.7)	22 (41.5)	12 (23.5)	0.040
	No	70 (67.3)	31 (58.5)	39 (76.5)	
Challenges for CGP					
	Turnaround testing time	59 (56.7)	38 (71.7)	21 (41.2)	0.008
	Lack of access	22 (21.2)	5 (9.4)	17 (33.3)	
	Sample issues: shipping	20 (19.2)	9 (17.0)	11 (21.6)	
	Costs	3 (2.9)	1 (1.9)	2 (3.9)	
Confident interpreting CGP results?					
	Yes	79 (76)	45 (84.9)	34 (66.7)	0.025
	No	25 (24)	8 (15.1)	17 (33.3)	
Confident treating stage IV oncogenic-driven LUAD?					
	Yes	88 (84.6)	47 (88.7)	41 (80.4)	0.184
	No	16 (15.4)	6 (11.3)	10 (19.6)	
Confident treating TKIs’ AE?					
	Yes	85 (81.7)	44 (83)	41 (80.4)	0.460
	No	19 (18.3)	9 (17)	10 (19.6)	
Confident provider					
	Yes	69 (66.3)	41 (77.4)	28 (54.9)	0.013
	No	35 (33.7)	12 (22.6)	23 (45.1)	

AE, adverse event; CGP, comprehensive genomic profiling; LUAD, lung adenocarcinoma; TKI, tyrosine kinase inhibitor.

Overall, the three confidence domains analyzed were statistically associated (interpreting CGP and treating driver-positive LUAD, *p* = 0.003; interpreting CGP and managing TKI adverse events, *p* < 0.001; treating driver-positive LUAD and managing TKIs, *p* < 0.001). Nevertheless, we found two relevant discordances within the confidence domains. First, of the 104 participants, 19 (18.2%) stated that they are not confident in interpreting CGP but are confident in treating oncogenic-driven tumors or managing TKIs’ adverse events. Most of these providers generally treat fewer *EGFR*-mutated patients (*p* = 0.02), or *ALK*-translocated patients (*p* = 0.06), than providers stating confidence in interpreting CGP. Second, nine participants (8.2%) reported confidence in interpreting the genomic testing results but lacked confidence in treating oncogenic-driven lung cancer or managing tyrosine kinase adverse events. Details are found in Supplementary Figure 1.

Confident providers were more likely to request CGP for LUAD than nonconfident providers (66% versus 34.2%, respectively, *p* = 0.01). They were also more likely to have more than 10 years of experience practicing oncology (49% versus 31%, *p* = 0.06) and have higher clinical loads of patients with lung cancer in general (34.8% versus 14.3%, respectively, *p* = 0.02), of both *EGFR-*mutated (24.6% versus 8.6%, respectively, *p* = 0.040) and *ALK-*translocated patients (30.4% versus 8.6%, respectively, *p* = 0.008) compared with nonconfident providers. Confident providers also tend to have increased in-house genomic testing availability than nonconfident providers (38% versus 23%, respectively, *p* = 0.09).

Unadjusted associations between participants’ characteristics and their confidence are depicted in Table 2. Systematic requesting CGP to stage IV LUAD revealed significant associations with provider’s confidence (adjusted OR = 0.35, 95% confidence interval: 0.14–0.84, *p* = 0.028) after controlling for the region of practice, lung cancer clinical load, time practicing oncology, and availability of in-house testing. Details are found in Table 2.

**Table 2:** Univariable and Multivariable Regression Revealing the Association Between Providers’ Confidence and Other Variables of Interest

		Univariable Analysis			Multivariable Analysis		
Variables of Interest	Categories	OR	*p* Value	95% CI	OR	*p* Value	95% CI
Brazilian region	Southern regions	2.74	0.04	1.01–7.51	4.01	0.014	1.33–12.06
	Others						
Lung cancer clinic load	<10 patients (ref)	3.20	0.033	1.09–9.31	2.94	0.067	0.93–9.31
	≥10 patients						
Time practicing oncology	<10 y (ref)	2.12	0.085	0.90–4.98	1.80	0.226	0.69–4.67
	≥10 y						
In-house testing	Yes (ref)	0.49	0.132	0.19–1.23	0.57	0.308	0.20–1.65
	No						
Systematically request CGP to stage IV LUAD	Always (ref)	0.35	0.017	0.15–0.83	0.35	0.028	0.14–0.84
	Not always						

CI, confidence interval; LUAD, lung adenocarcinoma; ref, reference.

### Comprehensive Genomic Profiling

Among participants, 51 (49%) reported not requesting CGP for all patients with stage IV LUAD they see. Participants from the Southeast region tend to request it more frequently than participants from other regions (64% versus 45%, *p* = 0.039). In addition, medical oncologists who see more patients with *EGFR* mutations and AL*K* rearrangements, and those with in-house genomic testing available, request CGP more frequently (*p* = 0.004, *p* = 0.023, *p* = 0.04). Participants requesting CGP less regularly are more likely to have uncertainties on all three confidence domains evaluated (interpreting genomic results, *p* = 0.025; self-distrust managing driver-positive lung cancer, *p* = 0.003; and managing TKI adverse events, *p* = 0.016).

**Figure 2:**
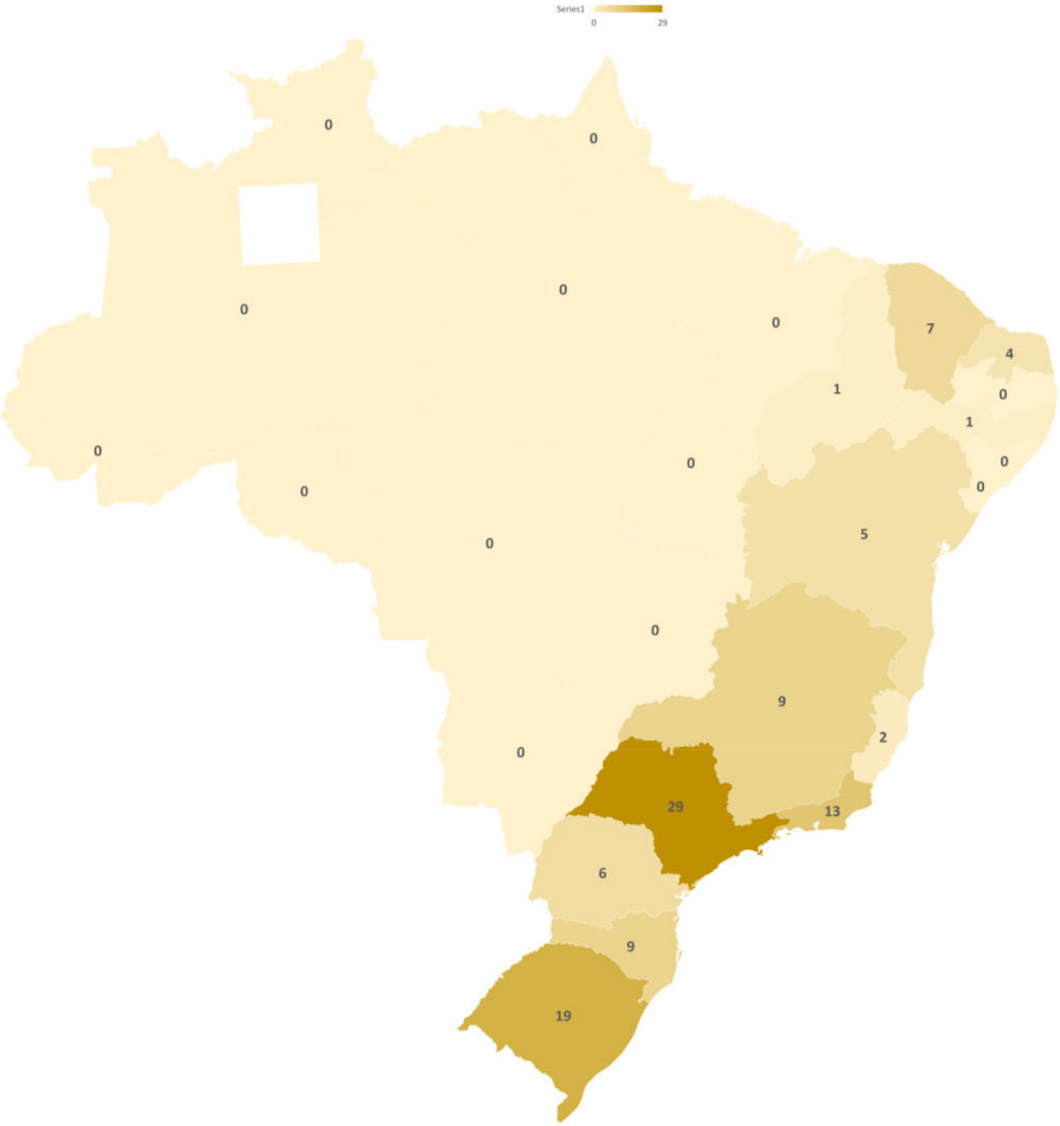
Brazil map illustrating the number of lung cancer clinical trials currently recruiting patients in each state of Brazil. The dashed line reveals the limits separating Southeast and South Brazil from other regions. Adapted from https://clinicaltrials.gov/ct2/results?cond=Lung+Cancer&term=&cntry=BR&state=&city=&dist=.

Medical oncologists reported various detailed challenges for ordering CGP, including (1) excessively long turnaround time from sending samples for analysis and receiving results back, delaying decision-making (56.7%); (2) lack of access to treatments or clinical trials directed to the genomic alterations eventually detected in sequencing tests, discouraging requests (21.2%); (3) challenges regarding the sample, including poor quality samples and bureaucracy to send the sample for analysis (19.2%); and (4) high sequencing costs (2.9%). The frequencies of these challenges varied by the region of Brazil; although turnaround time was the most frequent challenge found in all states and regions, the state of São Paulo was significantly less affected than other states (44.2% versus 65.6%, *p* = 0.025). In addition, we found that participants from the North and Mid-West regions tend to have more difficulty accessing drugs and including patients in clinical trials (*p* = 0.06). To illustrate this information, we captured lung cancer clinical trials open for recruitment in Brazil in Figure 2. It is visible that Northern regions lack clinical trials compared with the Southern regions.

All participants reported at least one challenge in the process of requesting CGP or getting CGP results. The genomic sequencing challenges differed between confident and nonconfident providers without statistical significance. Turnaround time was the greatest challenge for 61.4% of the confident providers and to 47.1% of the nonconfident providers. In comparison, lack of access to drugs or clinical trials was the greatest challenge for 15.7% and 32.4%, respectively (*p* = 0.149). The description of the collected variables is detailed in Table 1. We had one qualitative comment, illustrating very well all the challenges together: “I order CGP for all the patients I see in my private clinic, however, for public health care patients, I order only liquid biopsy for *EGFR* because of logistical issues and lack of access to target-treatments.”

Next, we looked at the challenges reported by the 51 participants who did not request systematic testing. They reported greater difficulty in accessing medications and clinical trials than participants who requested systematic broad genomic sequencing (33% versus 9%, Fisher’s exact test, *p* = 0.007). The challenges encountered in other country regions seem balanced, as depicted in Table 1.

## Discussion

In this study, we focused on providers’ confidence in interpreting genomic findings and managing oncogenic-driven LUAD (treating patients and controlling side effects) and the frequency of use of genomic test. Our data suggest that providers’ confidence is a strong predictor of genomic test use and that even in a setting without cost barriers, challenges such as physicians’ awareness and long test turnaround time negatively affect genomic test use.

Overall, we found a high rate of confident providers, with 67% stating confidence across all three domains. Providers’ confidence was correlated with the availability of in-house genomic testing, more than 10 years of oncology experience, and high volumes of patients with lung cancer in general, and patients with *EGFR*-mutated and *ALK*-rearranged lung cancer. In other studies, surveying physicians treating multiple tumor sites, providers’ confidence tends to be above 75% and correlated with confidence in using test results in patient care.^12^^,^^13^ In a cohort including hematological and solid malignancies, providers with higher confidence in interpreting genomic findings had a sixfold higher chance of testing more patients than those with a low confidence.^9^ Similarly, a recent study including 1281 oncologists practicing in various regions of the United States also found that confidence in interpreting genomic findings was positively associated with test use.^13^ These studies analyzed providers’ confidence in interpreting genomic findings as an isolated variable. In contrast, we analyzed genomic confidence in conjunction with provider self-reported confidence in treating oncogenic-driven lung cancer and in managing TKI adverse events. Thus, the variable providers’ confidence in our study brings essential features to physicians practicing lung cancer oncology currently, with both genomic understanding and clinical abilities.

In line with previous literature, 24% of the study participants lacked confidence in interpreting genomic test results. Understanding the basic principles and limitations of CGP used in clinical practice should be a priority for oncologists involved in lung cancer care. To a certain extent, pathologists and thoracic surgeons should also have basic training on precision oncology in lung cancer, optimizing interpretation and ensuring that patients receive the best care.^14^ Multidisciplinary inputs, such as Molecular Tumor Boards and Clinical Supportive Tools, may also aid genomic translation (read depth and coverage of different tests, variant calling, variant meaning, etc.) to clinical practice helping to implement lung cancer precision oncology in clinical practice.^15^, ^16^, ^17^

Most guidelines endorse somatic genomic testing in LUAD because of the potential to affect therapy: approximately 60% of patients have genomic driver alterations, and several studies reveal that using genomic testing to implement guideline-concordant targeted treatment improves long-term outcomes in patients with oncologic malignancies.^18^^,^^19^ Nevertheless, extensive literature suggests that genomic test use is still far from ideal. As few as 15% of patients with LUAD treated in the community setting in the United States undergo CGP, with greater than 30 genes tested. Furthermore, less than 50% of patients with nonsquamous lung cancer undergo minimum testing of *EGFR*, *ALK*, *ROS1*, *BRAF*, and programmed death-ligand 1.^20^ Nonetheless, recent real-world studies point to an increase in genomic testing in the past years: observational data collected in the United States reveal an increase of patients ever tested by CGP from 28% in 2015 to 68% in 2020.^21^^,^^22^ The reasons for the lack of widespread CGP requisition include costs, unavailability of proper tissue for testing, and a perceived lack of benefit from the treating physician. Our analysis was performed in a scenario where cost is not a current limitation for tissue testing, as the LungMapping Consortium has sponsored FoundationOne testing for metastatic LUAD since 2019 in Brazil. Nevertheless, only 51% of the surveyed oncologists reported requesting CGP for all stage IV LUAD. Moreover, 22% of the oncologists stated that they request CGP for less than 40% of patients with metastatic LUAD, whereas 10% request it for less than 10% of patients with LUAD.

In our analysis, the most frequently reported challenges to proper tumor sequencing were as follows: (1) excessive turnaround time, (2) lack of access to affordable treatments or clinical trials discouraging test requests, and (3) sample issues, including poor quality samples and bureaucracy to send samples to analysis. These challenges are similar to those reported in previous studies performed worldwide, except for the cost barrier, because CGP is currently free of cost for LUAD in Brazil. Looking specifically at the challenges reported by the 51 participants who do not request systematic CGP, we found that they have more significant challenges with access to affordable medications and clinical trials than participants who systematically request CGP. One possible reason for this is that most of these oncologists work in the public health system, where access to standard medications is limited (Supplementary Table 1). For instance, *ALK*-targeted therapy and immunotherapies for LUAD are still unavailable in the Brazilian public health system, whereas *EGFR*-targeted treatment has only recently been incorporated into the Brazilian public health system.^23^ Cronemberger et al.^6^ reported that only approximately 54% of LUAD were tested for *EGFR* mutation in 2014 in Brazil; frequencies of testing were significantly lower for patients in the public health system (70% versus 52%). Thus, oncologists’ perceptions of the testing value to these patients individually are low. Understanding the need for reliable data and quality improvement research to assess the magnitude of oncogenic-driven LUAD in the local Brazilian reality is necessary so that oncologists value CGP even though the individual patient might not directly benefit from the result owing to lack of access to affordable treatments. Most oncologists in low- to middle-income countries lack formal training in research methods. This scenario is unlikely to change soon owing to a lack of proper research training in the medical graduation and oncology residency training curricula.^24^ A systematic effort to educate oncologists about the value of CGP and other molecular testing modalities focused on low- to middle-income countries should be part of a strategy of global organizations, such as the American Society of Clinical Oncology and European Society of Medical Oncology. In addition, as revealed here, oncologists in Brazil lack specific training in lung cancer and usually practice as generalists. As depicted in Figure 1, the density of oncologists per 100,000 Brazilians is low in many states, especially in the North, Northeast, and Mid-West regions, which are knowingly less economically developed. In contrast, it is higher in the most prominent economic regions, the South and Southeast. The low oncologist density in the Northern regions may be related to the lower confidence in managing lung cancer in these areas. Oncologists in the Northern regions are obliged to practice as generalists and are less accustomed to working in lung cancer clinical trials, as depicted in Figure 2. Altogether, these factors arguably result in lower confidence and lower CGP use. Solutions are urgent and need to be addressed by national organizations, encouraging oncologists to work in less privileged areas and empowering local leaders to improve the quality of care. In addition, national multidisciplinary molecular boards and improving access to targeted therapies are possible alternatives to lessen these geographic inequalities in Brazil.

The testing situation is somewhat different in early stage LUAD, where the standard of care is surgery with adjuvant or neoadjuvant chemotherapy. In this scenario, the value of testing is even less recognized by physicians. Nevertheless, in light of the recently published ADAURA trial, testing for *EGFR* mutation is necessary for stages IB to IIIA, as patients could be eligible for adjuvant osimertinib. Such testing is currently not routine, but this discussion should undoubtedly be addressed by global organizations as well.^25^

Our study has a few limitations. Our study represents a population-based sample of adult cancer providers treating lung cancer and affiliated with GBOT. Thus, our findings may not be generalized to all community centers, where providers may not necessarily be part of lung cancer societies. Our study may also be limited by nonresponse bias. As we shared the survey link by means of e-mail to GBOT members, we had a moderate response rate, lower than previously published physician surveys.^12^^,^^26^ It is important to emphasize that exposing oncologists’ confidence in managing lung cancer may cause discomfort and distrust. To minimize that, we have adopted a strategy to prioritize anonymity and avoid exposing respondents who do not want their confidence to be publicized. Therefore, we have not provided financial incentives to respondents nor verified if the e-mail had been received or called their offices to encourage participation. The disadvantage of preventing these strategies, common in opinion research, is lowering response rates, as found here. In addition, we asked physicians about their intentions, and it is possible that an intention-behavior gap plays a role in physicians’ actions. Finally, we evaluated physicians’ confidence with a novel unvalidated measure, limiting our ability to draw definitive conclusions.

In conclusion, providers with higher confidence in interpreting genomic findings and managing driver-positive patients with lung cancer request more CGP. In contrast, providers lacking confidence in interpreting genomic results or managing driver-positive lung cancer request CGP less frequently. More comprehensive data should be pursued to understand better the complexity of CGP testing, such as real-life databases, properly evaluating providers’ genomic knowledge, and combinations of patient and provider surveys encompassing social-health–related questions. Improved access to medications and clinical trials and promoting providers’ awareness are necessary to increase CGP use and promptly inform treatment decisions.

## CRediT Authorship Contribution Statement

**Aline F. Fares:** Conceptualization, Writing—original draft.

**Pedro H. Martinez, Pedro H. Farina, Isaac Bicalho de Souza, Narayana S. Paiva, Ligia F. Orlando:** Data curation.

**Tatiana Colombo:** Formal analysis.

**Daniel V. Araújo:** Writing—review and editing.

**Eldsamira Mascarenhas, Ana Caroline Z. Gelatti, Clarissa Baldotto, Mauro Zukin, Luiz Henrique Araujo, Clarissa Mathias, Gustavo Werutsky, Gilberto de Castro Jr.:** Writing—review and editing.

**Vladmir C. de Lima:** Writing—review and editing, Supervision.

## Supplementary Data

Supplementaery Data
