# Supplementary material for: Assessing Oncologists’ Attitudes Concerning Comprehensive Genomic Profiling in Stage IV Lung Adenocarcinoma in Brazil
Source: JTO Clin Res Rep. 2022 Aug 30;3(10):100402. doi: 10.1016/j.jtocrr.2022.100402 (PMC9526154; doi:10.1016/j.jtocrr.2022.100402)
Supplement: Supplementaery Data [file mmc1.docx]

**Table 1.** List of tyrosine-kinase-inhibitors and immune checkpoint inhibitors currently approved by the FDA for lung cancer treatment. In Brazil, initially, drugs must be approved by the Brazilian Health Regulatory Agency (ANVISA – Agência Nacional de Vigilância Sanitária). In private health coverage, oral medications are reimbursed only after their inclusion in the ANS (Agência Nacional de Saúde Suplementar) Rol, to be reimbursed. In the public health system – SUS – drugs must be approved by CONITEC to be included in the list of medications supplied by the public health system and provided to all Brazilians.

| **Disease** | **Drug** | **CONITEC** | | **ANVISA** | | **ANS Rol** |
| --- | --- | --- | --- | --- | --- | --- |
|  |  | **Approved?** | **Approval date** | **Approved?** | **Approval date** | **Is the below medication included?** |
| ***EGFR* sensitizing mutations** | Gefitinib | yes | November, 2013 | yes | July, 2011 | yes |
|  | Erlotinib | yes | November, 2013 | yes | November, 2012 | yes |
|  | Osimertinib | no | NA | yes, 1st line | April, 2018 | yes |
|  | Dacomitinib | no | NA | no | NA | no |
|  | Afatinib | no | NA | yes | June, 2017 | yes |
|  | Icotinib | no | NA | no | NA | no |
|  | Erlotinib/Ramucirumab | no | NA | yes | June, 2020 | NA |
|  | Erlotinib/Bevacizumab | no | NA | yes | December/2016 | NA |
| ***EGFR* exon 20 insertion** | Amivantamab | no | NA | yes | September, 2021 | NA |
|  | Mobocertinib | no | NA | no | NA | no |
| ***ALK* and *ROS1* fusions** | Crizotinib | no | NA | yes | February, 2016 | yes |
|  | Alectinib | no | NA | yes | January, 2019 | yes |
|  | Ceritinib | no | NA | no | NA | no |
|  | Lorlatinib | no | NA | yes, 1st line only | June, 2021 | yes |
|  | Brigatinib | no | NA | yes, 1st line only | May, 2021 | yes |
|  | Entrectinib | no | NA | no | NA | no |
| ***MET* exon 14 skipping mut** | Capmatinib | no | NA | yes | June, 2021 | no |
|  | Tepotinib | no | NA | yes | June, 2021 | no |
| ***RET* fusion** | Selpercatinib | no | NA | no | NA | no |
|  | Pralsetinib | no | NA | no | NA | no |
| ***NTRK* fusion** | Entrectinib | no | NA | no | NA | no |
|  | Larotrectinib | no | NA | no | NA | no |
| ***BRAF* V600E mutation** | Dabrafenib-Trametinib | no | NA | yes | June, 2021 | no |
| **No drivers, regardless of PD-L1** | Pembrolizumab | no | NA | yes | June, 2018 | NA |
|  | Atezolizumab | no | NA | yes | October, 2018 | NA |
|  | Cemiplimab | no | NA | yes | July, 2022 | NA |
|  | Nivolumab | no | NA | yes (in combination with ipilimumab and chemotherapy) | September, 2021 | NA |


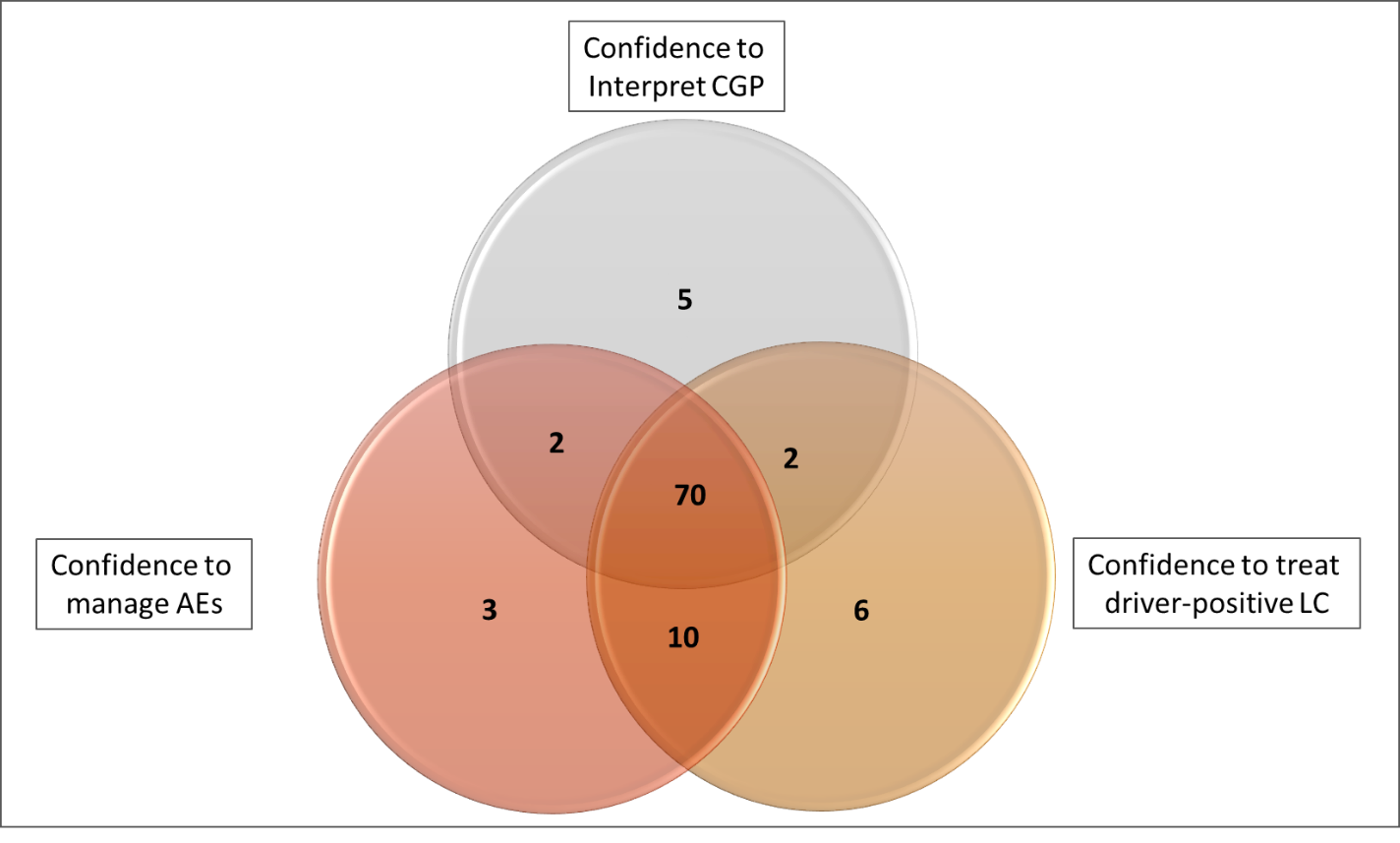


**Figure 1.** Venn diagram shows the relationships among the three confidence domains in the confidence variable.
